# Neonates in Ahmedabad, India, during the 2010 Heat Wave: A Climate Change Adaptation Study

**DOI:** 10.1155/2014/946875

**Published:** 2014-03-10

**Authors:** Khyati Kakkad, Michelle L. Barzaga, Sylvan Wallenstein, Gulrez Shah Azhar, Perry E. Sheffield

**Affiliations:** ^1^Department of Paediatrics, Smt S.C.L. General Hospital, Saraspur, Ahmedabad 380053, India; ^2^Department of Preventive Medicine, Icahn School of Medicine at Mount Sinai, New York, NY 10029, USA; ^3^Indian Institute of Public Health, Gandhinagar 380054, India; ^4^Ahmedabad Heat and Climate Study Group, Gandhinagar 380054, India

## Abstract

Health effects from climate change are an international concern with urban areas at particular risk due to urban heat island effects. The burden of disease on vulnerable populations in non-climate-controlled settings has not been well studied. This study compared neonatal morbidity in a non-air-conditioned hospital during the 2010 heat wave in Ahmedabad to morbidity in the prior and subsequent years. The outcome of interest was neonatal intensive care unit (NICU) admissions for heat. During the months of April, May, and June of 2010, 24 NICU admissions were for heat versus 8 and 4 in 2009 and 2011, respectively. Both the effect of moving the maternity ward and the effect of high temperatures were statistically significant, controlling for each other. Above 42 degrees Celsius, each daily maximum temperature increase of a degree was associated with 43% increase in heat-related admissions (95% CI 9.2–88%). Lower floor location of the maternity ward within hospital which occurred after the 2010 heat wave showed a protective effect. These findings demonstrate the importance of simple surveillance measures in motivating a hospital policy change for climate change adaptation—here relocating one ward—and the potential increasing health burden of heat in non-climate-controlled institutions on vulnerable populations.

## 1. Introduction/Background

Climate change is a growing international concern with current and future global effects. The Intergovernmental Panel on Climate Change (IPCC) reports unequivocal evidence for the warming of climate systems with a global mean temperature rising 0.74°C between 1906 and 2005, and the 11 years 1995–2006 have been reported as being among the 12 warmest years since 1850 [1]. These rising temperatures are especially disconcerting for urban areas.

Specifically, in urban areas the warming from climate change increases the risk of heat waves by compounding the existing problem of urban heat islands—a result of urban areas being hotter than surrounding rural areas due to more impervious and heat absorbing surfaces that retain and subsequently reradiate incident solar radiation, less vegetation, and more local heat production [2, 3]. As such, climate change affecting these areas will have far-reaching consequences, as the proportion of the global population living in urban areas increases. For example, the urban proportion of the global population increased from 30% in the 1950s to over 50% in the early 2000s and, by the year 2030, 60% of the world's population is expected to live in cities, according to the United Nations (2001) World Population Prospects Revision Report [4].

One city of importance is Ahmedabad, India, with a population of 7.2 million [5]. Ahmedabad is the largest city in Gujarat and among the fastest growing cities in India [6]. Located in the arid Northwest region of India, Ahmedabad's warm, dry conditions often include heat waves. Ahmedabad's hot season typically runs from March to June, with an average monthly maximum of 38.8°C (101.8°F) during this period [7]. According to the India National Assessment, the region of Western Ghats where the city of Ahmedabad is located is projected to have mean annual temperatures of 26.8 ± 0.4°C–27.5 ± 0.4°C in the 2030s, an increase of 1.7-1.8°C with respect to the 1970s [8]. In May 2010, Ahmedabad experienced a record-setting heat wave which anecdotally resulted in increased all-cause mortality as temperatures locally reached as high as 46.8°C [9]. The health department of the Ahmedabad Municipal Corporation (AMC) reported that the total death toll from this heat wave was 47, with 29 deaths from heatstroke alone [10]. However, this was based on a preliminary analysis without a formal excess mortality assessment.

The informal death toll reported in Ahmedabad is unfortunately realistic or even an underestimate of the actual health impact from the heat wave according to studies of heat-health impacts elsewhere. Even high resource countries have significant health effects during heat waves. For example, in some northern US cities, the effects of heat have increased summer mortality by nearly 40% over baseline averages [11]. Also in the United States, heat waves cause more deaths annually than any other natural disaster [12].

Health effects from heat waves extend beyond just increased mortality. The U.S. Centers for Disease Control and Prevention lists the three most common types of heat-related illnesses (HRI) as heat cramps, heat exhaustion, and heatstroke [13]. These types of illness can occur when individuals are exposed to extreme heat and may lead to death if not properly diagnosed and treated [11]. The study of heat-related illness is a growing area and, thus far, has been primarily studied in occupational settings. For example, studies have found that workers exposed to extreme heat may be at risk of several types of heat-related illnesses [14]. However, the effects of extreme heat are not limited to occupational settings nor only heat-related illness. In a review looking more broadly at elevated ambient temperatures, defined in this review and for the purposes of this study as outdoor temperatures as measured by standard meteorological services and mortality studies from 2001 to 2008, there was an association with higher ambient temperatures and increased risk of death from respiratory, cerebrovascular, and cardiovascular diseases, including ischemic heart disease, congestive heart failure, and myocardial infarction across all ages, race, and gender [15].

Higher ambient temperatures are also associated with other types of illness besides heat-related illness. In a California study, high ambient temperature was significantly associated with preterm birth, regardless of maternal racial/ethnic group, maternal age, maternal education, or sex of the infant [16]. Importantly, certain groups such as younger mothers, Blacks, and Asians had an even higher association of negative outcomes and temperature indicating the potentially important role of socioeconomic factors. One review of epidemiological literature identified 20 studies that investigated seasonality of birth outcomes and reported statistically significant seasonal patterns. Most of the studies found peaks of preterm birth, stillbirth, and low birth weight in winter, summer, or both, which indicates the extremes of temperature may be an important determinant of poor birth outcomes [17]. Increasing ambient temperature has also been associated with infectious diseases such as diarrhea-associated morbidity in a 10-year Taiwanese study [18] and, indirectly, through changes in air quality, with respiratory illness [19]. Ambient temperatures are shown to be a universal hazard and concern, as they present various and vital implications to public health.

This epidemiologic literature indicates that infants are a population particularly vulnerable to the rising global temperatures. While keeping an infant warm enough to prevent hypothermia has been the focus of much neonatal work, preventing hyperthermia is also important [20]. Homeothermy requires a balance among heat production, skin blood flow, sweating, and respiration in such a way that changes in heat loss or gain from the environment are precisely compensated. Greater susceptibility of body temperature to ambient temperatures is to be expected in smaller organisms because of their large surface area to volume ratio, the relatively small insulating body shell, despite a higher percentage of brown adipose tissue in newborns, and the smaller body mass that acts as a heat buffer in large organisms [21]. Additionally, any pathological conditions such as malnutrition, as is widespread in India, or pulmonary disease can further exacerbate a baseline physiologic susceptibility. A review of data suggests that despite attempts to eliminate heat through adjustments in the peripheral circulation (narrowing central peripheral gradients) infants are not as able as older children and nonelderly adults to maintain set-point temperatures [19].

In addition, the difficulty neonates have in thermoregulation makes them a potentially vulnerable population during times of extreme heat. In previous reports on heat waves and high ambient temperature, very few studies have considered or found effects in infants and young children [22]. This is perhaps due to most studies being conducted using data from developed countries with likely higher rates of climate-controlled, indoor settings. A retrospective study of mortality related to a 2003 heat wave in France found moderate but significant excess mortality also occurred among male infants aged less than 1 year [23]. Another study done in California found increased mortality risk in infants 1year of age or less [24].

During a scientific workshop in Gujarat, India during the spring of 2011 focusing on heat and health [25], preliminary data highlighted the potential burden of disease on infants in non-climate-controlled buildings there, such as most homes and hospitals. Non-climate-controlled refers to the absence of a heating or cooling system that regulates temperature and humidity within a building. One energy survey estimated that only 129 hospitals have air-conditioning of the approximately 16,000 total hospitals across the country [26, 27]. A review of the literature—including both PubMed and Web of Science searches—revealed little work on the association of elevated ambient temperatures with morbidity or mortality in non-climate-controlled settings (see supplementary Annex 1 in Supplementary Material available online at http://dx.doi.org/10.1155/2014/946875). While absence of air-conditioning is a known risk factor for heat stroke and death among the elderly in residential settings and nursing homes in New York City [28], other institutional settings and health effects have not been studied. This study examines neonatal morbidity in a non-climate-controlled hospital setting during the May 2010 heat wave in Ahmedabad. Two primary questions of interest are (1) was there a relationship between high ambient temperatures and the rate of neonatal intensive care unit (NICU) admissions during the May 2010 heat wave in Ahmedabad? and (2) did the relocation of the maternity ward from top to ground floor have an effect on subsequent NICU admission rates?

## 2. Methods

### 2.1. Study Setting

**Figure 1:**
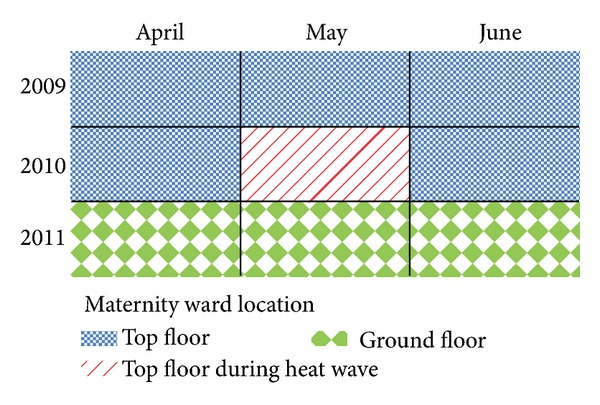
Nine-month study period, April–June 2009–2011, relative to the maternity ward hospital floor location.

This study is a retrospective review of hospital records for births and NICU admissions with a diagnosis for heat-related illness that occurred between April 1st and June 30th during the years 2009, 2010, and 2011 at the SCL General Hospital, serving a primarily low income population, in Ahmedabad, Gujarat, India. The data was obtained by manual transcription of the paper charts from the years of study. In 2009 and 2010, the maternity ward was on the fourth, highest, and reportedly hottest floor of the hospital, including May 2010 when the heat wave struck the city. After the 2010 heat wave but prior to the 2011 warm season of April, May, and June, the maternity ward was relocated to the ground and reportedly coolest level (Figure 1). Selection of the nine months enabled comparison with similar season months both before and after the 2010 heat wave event. Temporal matching such as the one used here is commonly used in heat wave analyses and helps to minimize confounding by population scale variables [29–31].

### 2.2. Data

For the nine months in study, the data included the number of daily births at the hospital, total daily NICU admissions, and daily NICU admissions for heat-related illness (i.e., heat-related admissions). NICU and heat-related NICU admissions included neonates—infants 0–28 days old —born outside of hospital and in-hospital transfers from maternity ward. The study outcome of heat-related illness was a diagnosis of exclusion defined as neonates admitted to the NICU with body temperature ≥38°C with any of the following signs or symptoms: refusal to feed, signs of dehydration, weight loss > 10% of birth weight, increased respiratory rate, convulsions, and/or lethargy. Exclusion criteria included evidence of septicemia, acute gastroenteritis, or central nervous system infection based on available cultures or labs.

Weather data was obtained in person by study author (KK) directly from the India Meteorological Department. Data included daily maximum and minimum temperatures and two daily relative humidity levels for the time period of April 1–June 30 for the years 2009, 2010, and 2011. Air pollution data was not available.

### 2.3. Analysis

To evaluate the effects of extreme heat on neonatal outcomes, we looked for an effect of maximum temperature and floor of the NICU on admissions for heat, as well as on total deliveries and non-heat-related NICU admissions. Humidity was included in preliminary models but ultimately excluded because no association was seen and from a policy perspective humidity is not included in the weather department's heat wave definition. First, graphical methods were used to suggest the type of function that would relate (heat-related) admissions to temperature. The preliminary visual evaluation suggested that the effect of temperature on admissions began at some point between 40°C and 42°C. We used Generalized Linear Models to evaluate the number of daily events, which we assumed followed a Poisson distribution. The variables used to predict this outcome were (i) maximum temperature per day, (ii) number of deliveries over the past three days, (iii) and whether the ICU was located on the top floor (in 2009 and 2010) or on a lower floor. For the effect of heat, we used segmented regression and specifically chose a horizontal line up to a certain temperature *T* and then for max⁡_*T* > *T* joined this segment with a line with slope *β*_3_. To further explore breakpoints, temperatures were selected from the 10th percentile of maximum temperature at 38°C, to the 90th percentile at 43.5°C for the temperature, *T*, in which the segmented line was allowed to slope upwards. The temperature of the “best fit” for the equation below, evaluated based on the log likelihood, was 42°C. We used proc genmod of SAS software, to estimate the parameters *α*, *β*_1_, *β*_2_, *β*_3_ in

(1)  Log[heat-related  admissions] =α+β1(births−7.4)+β2Floor for  max⁡_T<TC°,Log[heat-related  admissions] =α+β1(births−7.4)+β2Floor  +β3(max⁡_T−T)  for  max⁡_T≥TC°,

where births is the average number of births over the previous 3 days, floor = 0 for years 2009 and 2010 and floor = 1 for 2011, and max⁡_*T* is the maximum temperature in degrees Celsius. The same model was used for the secondary analysis of all NICU admissions, and a similar model was used for births except, obviously, the births term was removed.

### 2.4. Ethical Clearance

This study was approved by Smt. N.H.L. Municipal Medical College of Ahmedabad's Intramural Research Committee and the Program for the Protection of Human Subjects of the Icahn School of Medicine at Mount Sinai.

## 3. Results

**Figure 2:**
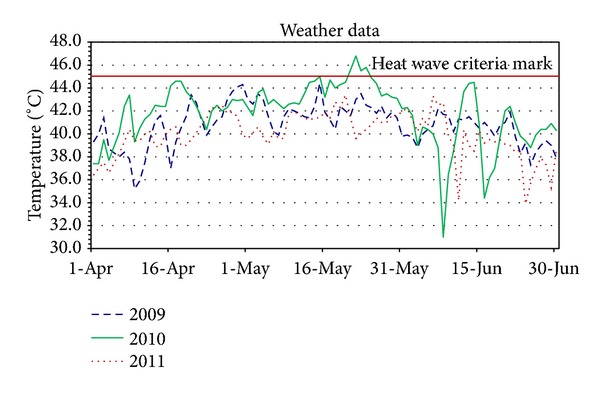
Maximum daily temperatures (degree Celsius) for the 9 months of study from India Meteorological Department's weather data. Heat wave criteria are either 45 degrees Celsius (line shown in red) or lower if daily maximum temperature is >4 degrees Celsius above the normal maximum daily temperature.

In the context of evaluating the effect of heat, we note that over the entire 9 months of study, daily maximum temperatures ranged from 31.0° to 46.8°C. The average maximum temperature for 2009 was 40.8°C, 41.8°C for 2010, and 40.3°C for 2011 (Figure 2). According to the India Meteorological Department criteria (heat wave definition used the “Climate of Ahmedabad” booklet (India Meteorological Department, January 2012) criteria: (a) when normal maximum temperature of a station is less than or equal to 40°C: (i) heat wave: departure from normal is 5 to 6°C and (ii) severe heat wave: departure from normal is 7°C or more. (b) When normal maximum temperature of a station is more than 40°C: (i) heat wave: departure from normal is 4 to 5°C and (ii) severe heat wave: departure from normal is 6°C or more. (c) When actual maximum temperature remains 45°C or more irrespective of normal maximum temperature, heat wave should be declared), the days of April 17 and 18 and May 13–15, 17, and 20–25 in 2010 qualified as a heat wave with daily maximum temperatures varying between 44.5° and 46.8°C. During these 12 days, there were 111 total births at SCL GH with an average of 8.5 per day, and a total of six neonates were admitted to the NICU with heat-related illness. Over the 273 study days, the temperature mean (SD) was 40.8 (2.3); the median temperature was 41.1°C, and the middle 50% of the days were between 39.5°C and 42.4°C, the 10th percentile was 38°C and the 90th 43.5°C.

Table 1 tabulates, for each of the 9 months of the study, the average maximum temperature, number of births to the study hospital, number of ICU admissions, and number of heat-related admissions. A total of 2,025 births occurring at the hospital were included in our analysis with an average of 7.4 births per day. Monthly total deliveries at SCL General Hospital ranged from 203 to 253 with an average of 225 deliveries per month. There were 554 total neonates admitted to the NICU, averaging 2 per day, with 36 of these being classified as admitted for heat-related illness. The number of total admissions to the NICU ranged from 51 to 82 per month, with an average of 61.5 admissions per month. The number of admissions to the NICU with a heat-related illness ranged from 1–13 per month. During the months of April, May, and June of 2010, there were 24 NICU admissions for heat, versus 8 and 4 in 2009 and 2011, respectively. When considering only babies transferred from the hospital's maternity ward into the NICU for heat-related illness, the number was 20 in the 2010 period versus 5 and 2 in 2009 and 2011, respectively

Descriptive statistics of infants admitted to the NICU with a heat-related illness, including gender, gestational ages, mode of delivery, low birth weight prevalence, age in hours at time of admission to NICU, >10% weight loss, fever, and maternity ward versus out of hospital transfer, can be found in Table 2.

In the models, in which the *T*, the turn for the segmented regression, that is, breakpoint, varied from the 10th percentile of maximum temperature at 38°C to the 90th percentile at 43.5°C, the effect of floor and of increases in temperature was statistically significant. As described in Section 2.3, the temperature of the “best fit” for our segmented regression model was 42°C. At this temperature (42°C), moving the maternity ward to a lower floor was associated with a predicted 64% reduction in heat-related admissions, 95% confidence intervals 3% to 89%. As for temperature association at this same breakpoint of 42°C, each increase in degree Celsius over 42°C was associated with a 43% increase in heat-related admissions, 95% confidence intervals 9.2% to 88%.

When examining non-heat-related NICU admissions instead of heat-related admission and then total number of deliveries, we found a similar direction of association though non-statistically significant results. For example, in the model looking at non-heat-related NICU admissions, controlling for floor and number of admissions, there was a 1% increase in admissions per degree increase over 40°C (*P* = .83) and a predicted 14% reduction due to moving the floor to a lower level (*P* = .13). For deliveries per day, adjusting for floor, there was an increase of 2% in the number of deliveries per 1% increase in degree over 40°C (*P* = .29).

Of note, we observe several outliers in which more heat-related admissions still occurred despite lower temperatures. Various factors including alterations in individual or community protective factors—conditions or behaviors that lower the effect of high temperatures on neonatal health—or other meteorologic variables could be responsible, but limited data sets prevented exploration.

## 4. Discussion

Analysis of the hospital and weather data found a relationship between ambient, or outdoor, temperatures and the number of NICU admissions for heat at a non-climate-controlled hospital in Ahmedabad, India. We also found moving the location of a maternity ward within hospital from the fourth and highest floor to the ground and reportedly cooler floor after the May 2010 heat wave showed a protective effect. Our data suggest that during the month of the heat wave, while the maternity ward remained on the top floor of the non-air-conditioned hospital, there was an increase in heat-related admissions to the NICU.

There are several possible reasons for the observed increase in NICU admissions for heat during the month of the 2010 heat wave. It could be that heat affects the infant in utero and causes him or her to be more vulnerable at birth due to either a younger gestational age (perhaps even just a few days) or physiologic stress from heat independent of the timing of the labor and delivery. Another possible reason for increased heat-related admissions is detection bias which perhaps shifted diagnosis of other illnesses toward heat. This possibility could not be ruled out completely because of lack of information about other diagnoses for other NICU admissions in our data set. Additionally, the findings from this study would have been strengthened if, when looking at the effect of the maternity ward location, we had patient data on a period of outdoor temperatures comparable to that of the May 2010 heat wave but from the ground floor location.

A previous study conducted on pregnant women in Barcelona found episodes of a relatively high heat index on the day before delivery was associated with a 2-day reduction in average gestational age at delivery and the most extreme heat condition was associated with a 5-day reduction, suggesting a dose response relationship [32]. While all babies admitted to the NICU for heat were listed as full term, the specific gestational age in weeks and days was not available to explore this further.

As previously mentioned, little research exists on the effects of high ambient temperatures and the vulnerable population of neonates. One area that has been looked at is the relationship between temperature and sudden infant death syndrome (SIDS). A possible role for hyperthermia in SIDS has been hypothesized because some victims are found in unusually warm environments, are warm and sweaty when found dead, are wrapped tightly in clothing or bedding, have a history of febrile illness before death, or have high rectal temperatures at examination or autopsy [33, 34]. In our study, none of the neonates died and so this was not examined. A larger dataset would permit exploration of these outstanding questions, which warrants the need for further study.

One of the primary limitations to this study is that the lack of descriptive information on non-heat-related cases limited the ability to examine individual level risk factors. Being able to examine other factors would possibly lead to evidence about protective effects and modifiable risk factors. For example, information is needed on neonates born at SCL General Hospital but not admitted to NICU, on all NICU admissions, and ideally on those brought into hospital following an outside of hospital birth. This information could be used to study other variables that may have contributed to heat vulnerability such as birth weight, socioeconomic status, mode of delivery, and breast or formula feeds. Three out of every five births in India take place at home [35]. Harsh environmental conditions and other negative factors could have a relatively large effect on birth outcome. We do know that some of the infants admitted to the NICU during the study period were born outside of the hospital, but we do not know their specific conditions nor their proportion of the total and thus are limited in the extent that we can explore the characteristics of these infants.

Additionally there are limitations to the exposure estimates used. Our meteorological data were for ambient, that is, outdoor, conditions only and were used as a proxy of indoor conditions. Direct indoor measurements would permit more precise characterization of the exposure. We can only estimate and infer based on what we know of indoor conditions in relation to outdoor temperature. The India Meteorological Department (IMD) records their temperature data near the airport located on the outskirts of the city. An urban heat island effect of 2–4°C has been reported in Ahmedabad, suggesting that residents may experience hotter temperatures than those reported [36].

Lastly, in terms of limitations, the highest temperatures occurred when the NICU unit was on the top floor. Thus there is a statistical problem in disentangling these two variables. We can find both temperature and floor significant even with the other in the model. However the co-linearity will increase the variability, and in interpreting the findings we should bear this dependence in mind.

Considering global health implications, it is important to note the difference in the heat wave criteria of Ahmedabad compared to that of a developed city in a temperate zone such as New York, NY. New York City and much of the northern United States issues alerts of excessive heat when the maximum heat index is expected to reach 37.8°C for a 24 hr period [37]. As previously mentioned, in Ahmedabad a “heat wave” is called when temperatures reach ≥45°C. According to a local media report, the 2010 heat wave was the first time the IMD issued a severe heat wave warning in Ahmedabad, cautioning people against sun stroke [38]. Repeated heat exposure has been known to decrease the sweating threshold, that is, a sensitization occurs in the course of repeated heat exposure rather than development of tolerance [39]. Those exposed to extreme ambient temperatures for extended periods of time will over time begin to sweat less. The sweating threshold shifts as it occurs during heat adaptation, accompanied by similar changes in the threshold temperatures for shivering and vasodilatation [40]. In Ahmedabad, the problem of repeated exposure to extreme heat is further compounded by a lack of easily available resources and public health awareness. Such climatic differences underscore the importance of local or regional data for best characterization of any given population's risk.

From a public health perspective, further research resources need to be directed to places where heat adaptation resources such as air-conditioners are scarce, yet the need is high. It is important for initiatives to be specific for a target audience and to consider heat wave criteria of location. In addition, public health solutions should look toward engineering and architectural design solutions such as central ventilation shafts used in older Ahmedabad buildings but not commonly in newer building and light-colored surfaces on rooftops to help with cooling on the top floors of buildings. There is a growing body of work regarding the energy savings from such relatively simple technologies [41], but the health effects of such interventions in non-climate-controlled buildings have not been well documented.

Recommendations for improved future research include a longitudinal study with descriptives collected on all data subjects. Improved data would include temperature measurements taken from inside the maternity ward and birth information from those brought into the hospital from outside births. In order to explore in more depth the preceding questions regarding protective factors or modifiable risk factors and the vulnerabilities of the fetus in utero versus the neonate in the prenatal period, additional epidemiologic studies, and ultimately a randomized controlled trial with comparable hospitals and control groups would add to the understanding of the effects of hospital cooling interventions.

## 5. Conclusion

This preliminary study supports the hypothesis that neonatal morbidity increases in non-climate-controlled settings during periods of extreme high ambient temperatures, applying evidence from study in Ahmedabad, India. Analysis of the maternity ward's location revealed both the detrimental effects of high outdoor temperatures and the positive health effects of a simple hospital level intervention. These findings demonstrate the importance of simple surveillance measures in motivating a hospital policy change toward climate change adaptation—here relocating one ward—and the potential, as yet little studied, increasing health burden of heat in non-climate-controlled institutions serving vulnerable populations. The health effects of climate change are a growing international concern that may have dramatic implications for the health of future generations. Newborns and pregnant women comprise some of our most heat-vulnerable populations and thus could be among those most affected by climate change. We emphasize the need for further research on climate change effects on and viable adaptation strategies for such heat-vulnerable populations.

## Supplementary Material

Supplementary material includes a list of search terms and search engines as well as specified dates that were used in the literature review that accompanied this study.

## Figures and Tables

**Table 1:** Average maximum temperature (Max._temp.), number of births to the study hospital, number of Neonatal Intensive Care Unit (NICU) total admissions (ICU_Adm.), and number of heat related admissions to the NICU (Heat Adm.) by month.

	April	May	June
2009	Max._temp. = 40.2	Max._temp. = 41.9	Max._temp. = 40.1
	Heat Adm. = 5	Heat Adm. = 1	Heat Adm. = 2
	ICU_Adm. = 82	ICU_Adm. = 61	ICU_Adm. = 59
	Births = 246	Births = 225	Births = 230

2010	Max._temp. = 41.7	**Max._temp. = 43.8**	Max._temp. = 39.9
	Heat Adm. = 3	**Heat Adm. = 13**	Heat Adm. = 8
	ICU_Adm. = 51	**ICU_Adm. = 75**	ICU_Adm. = 58
	Births = 203	**Births = 253**	Births = 209

2011	Max._temp. = 39.7	Max._temp. = 41.0	Max._temp. = 29.1
	Heat Adm. = 1	Heat Adm. = 2	Heat Adm. = 1
	ICU_Adm. = 52	ICU_Adm. = 53	ICU_Adm. = 53
	Births = 206	Births = 228	Births = 225

Bold type represents the values during the month of May 2010 when the heat wave event that triggered this study occurred.

**Table 2:** Descriptive statistics of neonates admitted to NICU with heat diagnosis.

Neonates admitted to NICU with heat diagnosis	2009, *n* = 8	2010, *n* = 24	2011, *n* = 4
Gender, male	100% (8)	54% (13)	50% (2)
Gestational age, full term	100% (8)	100% (24)	100% (4)
Mode of delivery, cesarean	37.5% (3)	25% (6)	0
Low birth weight, <2500 gm	50% (4)	37.5% (9)	0
Age (in hours) at admission, mean	80	67	140
Weight loss > 10% of BW	25% (2)	42% (10)	75% (3)
Transferred from within same hospital	62.5% (5)	83.3% (20)	50% (2)

*Counts are from the months April, May, and June of the corresponding year.
