# Supplementary material for: Neonates in Ahmedabad, India, during the 2010 Heat Wave: A Climate Change Adaptation Study
Source: J Environ Public Health. 2014 Mar 10;2014:946875. doi: 10.1155/2014/946875 (PMC3964840; doi:10.1155/2014/946875)
Supplement: Supplementary file 1 — Supplementary material includes a list of search terms and search engines as well as specified dates that were used in the literature review that accompanied this study. [file 946875.f1.pdf]

Annex 1

Details of literature review initially performed October 2011 and updated January 2013.

Search terms	Infant, neonate, newborn, low birth weight, premature birth, child, prenatal exposure, heat, ambient temperature, hot temperature, heat stress disorder, body temp changes, skin temperature, climate change
Search engines	Pubmed, Scopus, Web of Science, Google Scholar
